# Learning in Virtual Reality: Bridging the Motivation Gap by Adding Annotations

**DOI:** 10.3389/fpsyg.2021.645032

**Published:** 2021-03-24

**Authors:** Andrea Vogt, Patrick Albus, Tina Seufert

**Affiliations:** Department Learning and Instruction, Institute of Psychology and Education, Ulm University, Ulm, Germany

**Keywords:** virtual reality, signaling, intrinsic motivation, multiple representations in multimedia learning, coherence formation, mental models

## Abstract

One challenge while learning scientific concepts is to select relevant information and to integrate different representations of the learning content into one coherent mental model. Virtual reality learning environments (VRLEs) offer new possibilities to support learners and foster learning processes. Whether learning in VR is successful, however, depends to a large extent on the design of the VRLE and the learners themselves. Hence, adding supportive elements in VRLEs, such as annotations, might facilitate the learning process by guiding attention and supporting the selection of relevant information. Additionally, the mapping of pictorial and verbal information is eased by these annotations. The beneficial effect of annotations is highly dependent on learners' intrinsic motivation as intrinsic motivation while learning also affects the information selection and visual search patterns. In our experimental study (*N* = 61), we compared two conditions: learning in a VRLE with or without annotations. We measured the learning outcome on three different levels (knowledge, comprehension, and application). Additionally, we investigated intrinsic motivation as a moderator for the effect of annotations on learning outcome. We found no significant main effect of annotations on learning outcome. The moderating effect of intrinsic motivation for annotations on the overall learning outcome was significant. Our results imply that learners are either intrinsically motivated or need additional support by annotations as these support the selection of relevant information in the VRLE and therefore enable them to learn successfully. Which type or quantity of annotations supports learning processes best needs to be explored in future research.

## Introduction

Many different approaches to designing learning environments and supporting learners exist. Particularly, for science, often abstract representations of the learning content (e.g., in textbooks) challenges the learners to process, translate, and include concepts into their mental model (Rapp, 2007). As learning scientific concepts is *per se* challenging, the learners need to be supported by an appropriate presentation of the learning content. Demonstrations are such an appropriate form, which allows learners to explore scientific processes, such as chemical reactions. However, demonstrations cannot be used in all cases. Some processes might occur on a micro level and may not be observable (Zacharia, 2007; Akçayir et al., 2016). Some demonstrations are not applicable to a university setting because they need a complex technical setup, are too dangerous, or too expensive (e.g., Wilkerson-Jerde et al., 2015). In this case, new technologies, such as virtual reality (VR), can open the way to advanced education in different scientific domains (Potkonjak et al., 2016; Wu et al., 2020).

Using VR or 3D simulations to design learning environments offers the possibility to display a contextualized setting, but additionally provide the opportunity to analyze learning content in a different way (Fowler, 2015; Parong and Mayer, 2018; Radianti et al., 2020). These learning environments offer great opportunities for experimental learning and are described to be motivating, increase liking and engagement compared to conventional settings (Makransky et al., 2019; Di Natale et al., 2020; Klingenberg et al., 2020; Radianti et al., 2020). For example, invisible content can be made visible, and sensitive structures can be composed and decomposed flexibly. Furthermore, micro or macro phenomena, such as chemical reactions can be made visual in virtual reality learning environments (VRLEs) that cannot be observed in the physical world (Schott and Marshall, 2018; Han, 2020; Wu et al., 2020). Furthermore, potential risks can be overcome (Radianti et al., 2020). In contrast to virtual 3D animations, learners can immerse themselves in the VRLE. By immersion and the novelty effect of the VRLEs, learning processes might be additionally stimulated (Wu et al., 2020).

One challenge remains while learning science in a VRLE: Learners are usually confronted with different, multiple representations, such as text and pictures. The stereoscopic view of the VRLE impacts their perception and therefore might influence their cognitive learning processes (e.g., Paes et al., 2017). Mostly, next to the pre-dominant visual, pictorial presentation of the learning content, often auditory texts or short verbal labels or annotations are included (Mikropoulos and Natsis, 2011). All these different representations have to be understood and integrated into a coherent whole. For this integrative process of coherence formation, it is necessary to process the learning content deeply to identify and understand the relevant concepts and to integrate them (i.e., Bloom, 1956; Schnotz and Bannert, 2003). A common approach to facilitating such a deep-learning is to include highlights or signals to indicate relevant components of the learning content (Richter et al., 2016). Furthermore, the learners can be supported in finding corresponding elements (Gentner et al., 1993). For instance, adding annotations into the pictorial representation might help to connect corresponding information and highlight important aspects of the learning content (McTigue, 2009). Therefore, annotations might support and activate the learners, induce deeper cognitive learning processes, and foster coherence formation (Mayer et al., 1995).

Based on instructional design research, we know that these effects depend on the learner and their aptitudes (Seufert, 2003). Adding, for example, annotations might support learners with lower prior knowledge, and might cause interference for learners with high prior knowledge (Kalyuga, 2009). Hence, learners' aptitude and state need to be considered. Factors such as intrinsic motivation might also have a substantial impact on invested resources, as well as on the cognitive processing of the learning material (Moreno and Mayer, 2007; Baranes et al., 2014; Eseryel et al., 2014; Miranda and Palmer, 2014). Motivated learners show efficient visual search patterns which help them to learn successfully. Hence, annotations support learners with low motivation as they induce a more effective visual search pattern and consequently learning outcome is expected to be increased (Eseryel et al., 2014; Miranda and Palmer, 2014).

Recent studies compared VRLEs to other learning settings (e.g., Parong and Mayer, 2018; Makransky et al., 2019) and corroborate that VRLEs could be used for learning. However, these previous approaches and publications have not considered how VRLEs should be designed in order to support learning and the necessary cognitive processes. Most notably, they have not taken into account for whom VRLE should be designed in which way, particularly depending on learners' motivational state. Overall, the question of how a VRLEs needs to be designed to foster deep processing of the learning content, and how to support coherence formation depending on learners' motivational state, has not been answered yet. Therefore, the aim of the present study was to investigate the effect of annotations and their interaction with intrinsic motivation in VRLE on learning.

## Theoretical Background

The beneficial effects of annotations, the learners' intrinsic motivation, and the interplay of these factors will be addressed. To answer the question of how VRLEs should be designed for complex scientific content to foster learning and coherence formation, it is worth having a closer look at the relevant models that describe cognitive processes underlying learning with multiple representations.

### Cognitive Processes While Learning With Multiple Representations in Virtual Reality

One theory to describe learning processes is the *Cognitive Theory of Multimedia Learning* (CTML; Mayer, 2014). One assumption of this theory is that information is processed *via* two different channels (auditory and visual). It states that information is coded in two memory systems: the visual and the verbal system. This idea is based on the *dual-code theory* proposed by Paivio (1990). Thus, it is assumed that two separate mental models are built from these two information sources. At the end of the learning process, these two different mental models are integrated into one coherent mental model. This happens under consideration of the individual prior knowledge stored in the long-term memory (Mayer, 2014). Another assumption is that working memory only has a limited capacity to process information. This goes along with the finding that working memory overload can occur when too much information has to be processed at the same time (Baddeley, 1992). Furthermore, the CTML assumes that processing information is an active process, including selecting, organizing, and integrating information. This reflects the active role of the learner and emphasizes that the learner has to invest certain resources to learn successfully.

To better understand the integration process of verbal and pictorial information into the mental model, the *Integrated Model of Text and Picture Comprehension* (IMTPC) delivers more insights (Schnotz and Bannert, 2003). The *descriptive branch* describes the processing of verbal and textual information, and the *depictive branch* describes the processing of pictures.

Verbal information is first processed sub-semantically, which results in an internal representation based on the text surface. This enables learners to simply process the information without a deeper understanding and allows them to recall simple definitions or facts. Based on this superficially processed information further semantic processing takes place and results in a propositional representation or network. At this level, a learner would be able to understand the concepts. If learners need to mentally operate on the information and to apply it in other contexts, this propositional representation has to be translated into an analog representation, the mental model (Schnotz and Bannert, 2003). With this last step, learners switch from the descriptive to the depictive branch.

In contrast to this multi-level processing of textual representations, pictures are processed more directly *via* the depictive branch hence this branch is particularly important for learning in VRLEs. First, by perceptually processing a picture, the information is selected based on cognitive schemata. To semantically process this information, a picture comprehension process takes place by mapping visuospatial and semantic relations (Gentner et al., 1993; Schnotz and Bannert, 2003). Unlike the information processing in the descriptive branch, no additional translation of the processed information is needed to integrate the information because the created internal mental representation already has the same analog structure as the mental model (Schnotz and Bannert, 2003). Learners can also “read” and extract propositions from this mental model (i.e., they can again switch branches), in this case from the depictive to the descriptive side. Thus, there is an interplay between the descriptive and the depictive branch, and text and picture information can be integrated and mapped onto each other. However, neither the IMTPC nor the CTML describes in detail how this mapping and integration process takes place. Based on theories on learning with analogies or learning with multiple representations, these *mapping processes* can be distinguished into element-to-element and relation-to-relation mapping processes (Gentner et al., 1993; Seufert et al., 2007). Whereas element-to-element mapping processes enable the learner to connect the learning content on a superficial and syntactic level, relation-to-relation mapping processes refer to finding similarities on the semantic level (Seufert et al., 2007). Therefore, relational mapping provides support for developing a global understanding of the content, whereas element-based mapping supports connecting different components on a rather superficial level.

To deduce the different levels of processing, Bloom's (1956) taxonomy is often used to measure learning outcome in a differentiated manner. Questions aiming at the first level, the *knowledge* level, refer to the recognition or recall of facts, terms, or basic concepts without necessarily understanding what they mean. Hence, this reflects the superficial and sub-semantic processing of learning content. The next level, the *comprehension* level, involves semantic processing of the learning content. It refers to concepts or relational structures of single facts and can be derived when learners are for example, organizing them, describing them in summary form, or explaining the main ideas in their own words. For example, local relationships between the individual components can already be derived or concepts can be contrasted at this level. The next level of Bloom's taxonomy already assumes that a correct and coherent mental model has been formed. At the *application* level, learners can for example, further decompose the learning content into components and determine how different parts interact with each other. Furthermore, learned basic principles can be transferred to other use cases.

Based on these models, and particularly based on the assumption of IMTPC that pictures can be processed more directly, VRLEs seem to be a very promising approach to displaying complex scientific content. VRLEs are visual worlds consisting of mostly pictorial representations, thus, they support learners more directly in building a coherent mental model. Even content that is invisible in the real world can be drafted in the VRLE and can be used as a scaffold for constructing a mental model. When a brief scaffold based on a pictorial representation is constructed, adding further details to the mental model through the descriptive branch is a more simple and efficient way to construct a coherent mental model than vice versa (Schüler et al., 2015). The IMTPC implies that the process of building a coherent mental model out of textual and pictorial information is not a simple one, and consumes cognitive resources of the learners (Schnotz and Bannert, 2003). Therefore, the question arises as to how learners can be supported by the design of the learning environment to foster coherent formation processes, especially in VRLEs.

### Fostering Learning With Multiple Representations in Virtual Reality

In VR, the content is mainly displayed visually and the learners are confronted with highly salient visual or pictorial information. This perceptually demanding setting might lead to just-in-time processing, and learners might not be able to select and organize relevant information into a coherent mental model (Renkl and Scheiter, 2017). Therefore, an adequate design of the VRLE is one crucial precondition for learning success.

Two different approaches to support learners by the chosen design could be used: One approach would be to guide learners' attention to relevant aspects in the pictorial representation by displaying visual cues. Cueing refers to guiding attention by non-content means such as coloring or arrows (e.g., Mautone and Mayer, 2001; De Koning et al., 2007). However, this approach would guide learners' attention but would not provide additional support to map the auditory text to the given animation. Another approach is to use textual annotations to support both, selecting and integrating relevant information (Mayer et al., 1995). For example, the positive effect of annotating pictorial representations in learning scientific content was described by McTigue (2009). Learners with the additional textual annotation that labeled important components in the pictorial representation had significantly higher comprehension scores compared to controls without such annotations. Moreover, Mayer and Gallini (1990) found positive effects of annotations if these referred to parts and steps of scientific concepts. Considering their function, inserted annotations could be an effective mean to support learning processes in VRLEs. The three functions of annotations in the present study derive from the following facts: Annotations are salient, provided just-in-time, exactly at the place where they are needed, and repeat relevant information.

First, annotations function as signal as they are salient and appear just-in-time. Based on their salience, Mayer et al. (1995) states that “*annotated illustrations can serve as a signal that help readers select relevant words and images*” (p.40). Such signals guide attention toward certain aspects of the learning content and reduce unnecessary visual search and thus improve learning outcome (Richter et al., 2016). Hence, when learning in VRLEs, annotations can be used to emphasize certain components of the VR animation. The effect of signaling in this environment could be additionally strengthened when these annotations appear simultaneously to the corresponding narration, just-in time when learners should listen to the auditory text and combine this with the corresponding visual entity in the VR animation.

The second function is that annotations support mapping and integration processes, as they do not only appear just-in-time but also just-in-place (Gentner et al., 1993; Mayer, 2014). Based on this spatial contiguity, annotations signalize which aspects need to be mapped and integrated (Mayer and Fiorella, 2014). Learners' attention is drawn to the auditory text, the corresponding visual entity and a verbal label for this entity. Thus, the mapping process is eased as learners do not need to invest cognitive resources to find the corresponding elements (Mayer et al., 1995).

The third function is repetition. The textual annotation repeats crucial terms of the auditory text and is also redundant to the pictorial entity of the VR animation. Thus, based on the redundancy principle one might assume that such a repetition is irrelevant and learners could be distracted or extraneously overloaded (Kalyuga and Sweller, 2014; Mayer and Fiorella, 2014). However, as annotations usually do not display the same words as the narration but were only short labels, they can function as coherent information which adds to understanding (coherence principle; Mayer, 2005). Additionally, it is described that particularly learners with low prior knowledge benefit from redundant information (Adesope and Nesbit, 2012). This repetition of important aspects leads to higher learning outcome on the knowledge level (Paivio, 1990).

To sum up, the positive effect of short textual annotations on the learning process has already been explored in classical multimedia settings. Prior findings indicate that performance improvement can be found for recall of conceptual information [see also Boers et al. (2017)] and on higher levels of learning outcome, such as transfer or comprehension tests (McTigue, 2009; Mason et al., 2013). However, so far there have been no empirical studies examining the effects of annotations in a VRLE.

Despite the fact that the design of learning environments might support learners to process the information more effectively, there is one additional factor that might moderate these supporting effects, namely the learner him- or herself.

### Individual Requirements for Learning Success in a Virtual Reality Learning Environment

Aptitude-treatment interaction describes that while designing treatments for learners, one needs to consider their aptitudes. One important aptitude is prior knowledge. Depending on learners' prior knowledge, their performance is either enhanced by additional help or they are able to compensate for requirements of the given learning setting without additional help (e.g., Seufert, 2003). Therefore, learners' prior knowledge has often been taken into account when analyzing the impact of supporting conditions. However, as the effects of prior knowledge are well-researched, we want to address motivation as an additional important factor of the learner, which might be especially relevant in VRLEs. Based on the assumptions of the CTML, further development of the model reflects the importance of affective processes of learning: The *Cognitive Affective Theory of Learning with Media* (CATLM; Moreno and Mayer, 2007) postulates that cognitive processes are influenced by affective states, for instance by motivation. Hence, learning performance might be moderated by motivational states.

Researchers agree that learners who are motivated are more likely to persist on their task, and are more willing to engage and to expend effort for the required task completion (Di Serio et al., 2013). Some previous findings have suggested that using VRLE fosters learners' intrinsic motivation when these materials are compared to conventional learning materials, such as textbooks (Wu et al., 2020). Intrinsic motivation refers to doing something (e.g., learning) because it is inherently interesting, or the learner perceives the process as enjoyable. It is described as fostering learning and results in high-quality learning, as well as fostering creative processes (Ryan and Deci, 2000). Even for short learning tasks, intrinsic motivation is crucial for learning successfully (e.g., Fransson, 1977).

In a game-based virtual learning environment, Eseryel et al. (2014) uncovered an interaction effect of design and intrinsic motivation on learning by complex problem-solving. To explain this complex interaction of cognitive and motivational factors, underlying visual information processing needs to be considered. Previous studies imply that a higher intrinsic motivation goes along with a more efficient visual search pattern of the given task (Baranes et al., 2014; Miranda and Palmer, 2014). Hence, selection of relevant information is more efficient. As VRLEs are highly visually demanding, efficient visual search patterns are crucial for learning success. Therefore, particularly intrinsic motivation might have an impact on early information processes while learning and may have an impact on further cognitive processing of the learning content. Thus, being intrinsically motivated might have the same supportive effect on learning as annotations, namely an improved or eased visual search and a deepened learning process. Hence, the question arises as to whether both approaches—annotations as a design feature and motivation as a learner feature—might compensate for each other.

### Present Study

We aimed to gain further insights into how to design VRLEs for learning scientific concepts by using multiple representations appropriately. We investigated whether adding textual annotations has a positive effect on different levels of learning outcome (knowledge, comprehension, and application) while considering learners' aptitudes. Annotations, due to their two supportive functions (signaling and mapping), aimed to facilitate the construction of a coherent mental model.

Based on our postulated assumptions, we raise the following research questions:

(Q1) *Do annotations have a positive impact on learning outcome?* Previous studies showed beneficial effects of adding annotations on different levels of learning outcome (e.g., Mayer and Gallini, 1990; McTigue, 2009; Mason et al., 2013). Due to their signaling nature, annotations are expected to support the learner on a superficial level because they facilitate visual search and guide attention (McTigue, 2009; Ozcelik et al., 2010). Additionally, the annotations used in the present study are assumed to facilitate element-to-element mapping processes because they did not include additional information about processes or relations of the learning content. They simply labeled the visually displayed learning content with one or two words. Therefore, the used annotations simply repeated certain aspects and highlight connections between the textual label and the visual entity (Seufert et al., 2007). Both functions of annotations are expected to have a significant impact on learning outcome on the knowledge level (Mayer and Gallini, 1990; Boers et al., 2017). Based on the taxonomy of Bloom (1956), the different levels of learning outcome are characterized as hierarchical. Hence, the chosen annotations might also increase the learning outcome on a higher level, but not as much as on the knowledge level (Broadbent, 2017). Based on these discussed underlying cognitive processes, our expectations are as follows:

We hypothesize a significant and beneficial effect of annotations compared to no annotations in the VRLE considering the three levels of learning outcome: knowledge, comprehension and application (H1a). The largest positive effect of annotations on learning outcome is expected for knowledge (H1b).

Additionally, the intrinsic motivation in combination with the design of the VRLE might be crucial for learning successfully (Reynolds and Weiner, 2003; Moreno and Mayer, 2007). Therefore, our second research question is (Q2): *Does intrinsic motivation moderate the beneficial effect of annotations on learning outcomes?* As outlined earlier, intrinsic motivation and annotations are both expected to have a beneficial effect on learning outcome. Hence, these two factors might interact in a compensatory way: When learners are motivated, their visual search patterns are efficient and they learn successfully. Adding annotations might build a bridge for a motivational gap and induce a more effective visual pattern for less motivated learners, which should be reflected in an increase of the learning outcome.

We hypothesize that intrinsic motivation significantly moderates the relationship between the treatment factor annotations and learning outcome in the VRLE (H2).

## Materials and Methods

### A Priori Power Analysis

To estimate the necessary sample size, we performed an a priori power analysis. As no previous study investigated signaling in VRLE, we refer to a study that described signaling in a dynamic, system-paced animation for learning biology [De Koning et al., 2010; *f*^2^(V) = 0.35]. Based on the meta-analysis of Richter et al. (2016) this study reported rather large effect size (Cohen, 1988). Therefore, we performed our power analysis more conservative [*f*^2^(V) = 0.31; α =0.05; power = 0.95]. The needed sample size with the chosen effect size was *N* = 60 (G^*^Power 3.1.9.4.; Faul et al., 2009).

### Participants and Design

Our 67 participants were mainly university students in psychology as they were recruited as a part of an undergraduate course in psychology. Due to technical problems, we excluded 6 participants from further analysis. The remaining 61 participants (34.43% male) were aged between 19 and 52 years (*M*_*age*_ = 23.73; *SD*_*age*_ = 6.00). About 60% of the participants had at least once been in contact with VR in the past. We applied a between-subject design using a VR app and randomly assigned the participants to one of the two different design options: with (*n* = 33) or without annotations (*n* = 28). As a dependent variable, learning outcome was measured on three different levels: knowledge, comprehension, and application. In addition, learners' prior knowledge and the moderating effect of intrinsic motivation were assessed.

### Materials and Measures

In a short *demographic questionnaire*, participants were asked for their gender, age, educational level, their field of studies, and any prior experiences with VR glasses and applications.

Together with a domain-expert, we developed a *pre-test* for prior knowledge that aimed to measure domain-specific knowledge in science, with a special focus on relevant biological and chemical topics for seawater desalination. The test consisted of six open questions (e.g., “What is electrodialysis?”) and one closed question (allocation of different particles to particle types), with a maximum score of 20 points. Participants had to complete this knowledge pre-test prior to the learning unit.

**Figure 1:**
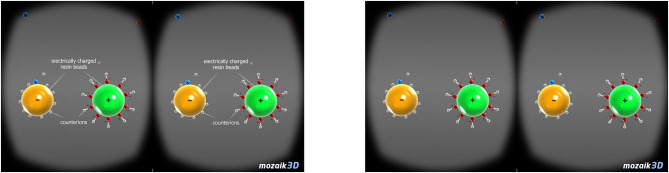
Virtual reality learning material with **(left)** and without **(right)** annotations.

To test the effect of annotations in VRLE, the pre-existing German version of the application for smartphones of MOZAIK education (Seawater desalination) was used (mozaWeb3D, 2018). In the respective learning unit, the desalination process for obtaining drinking water from seawater was shown. A special focus was put on different extraction methods and on general information about seawater and its characteristics. The participants saw visualizations of the learning content through a stereoscopic video in VR. Additional information and explanations were offered auditorially. The used application did not allow the learner to interact with and control the VRLE. The information displayed through the annotations in one of the two experimental conditions did not include further details, but rather simply emphasized certain important aspects of the learning content. The annotations were placed nearby the graphical representation to which they referred to (see Figure 1, mozaWeb3D, 2018). The application was run on smartphones that were combined with the VR glass ZEISS VR One Plus.

Furthermore, we developed a *post-test* for measuring the learning outcomes, which consisted of 13 questions on the three levels of Bloom (1956); knowledge, comprehension, application. To measure the learning outcome on knowledge level, six questions were developed including three open and three single choice questions (e.g., “Which process for seawater desalination is particularly environmentally friendly?”). For the comprehension level, four open questions were included (e.g., “Explain why seawater cannot be drunk in large quantities”) and for application we developed three open questions (e.g., “Which everyday object functions similarly to reverse osmosis?”). In the present study, the questions of the post-test aimed to examine different aspects of the learning content. Different questions referred to different concepts and processes. Hence, we expected no high internal consistency of the different questions. To ensure that learning outcome was measured in a rigorous way, we conducted two independent ratings based on a clear evaluation scheme. To analyze the inter-rater reliability, we used the Pearson product moment correlation coefficient, which revealed a very high consistency between the two ratings (*r* = 0.92, *p* < 0.001, *CI* = 0.86–0.96).

Intrinsic Motivation was measured by self-report with the respective subscale of the questionnaire of Lepper et al. (2005), which was based on the original questionnaire of Harter (1981). It consisted of three subscales: challenge, curiosity, and independent mastery. To ensure sufficient quality translation and re-translation has been performed and was compared to the original version. Items were slightly adapted to fit the VR learning context. The internal consistency of the intrinsic motivation scale was sufficient (α = 0.81; *CI* = 0.74–0.89).

To gain further insights into the subjective VR experience, the participants were asked to comment their impressions and on their well-being during the VR learning session at the end of the study.

### Procedure

The study took place in a realistic learning setting as part of the psychology course at the university. To ensure good data quality without disruption due to technical problems, the study was conducted in a standardized individual testing session with a trained investigator. At the beginning, participants were informed about the procedure of the study and signed an informed consent. All participants started the study by completing the demographic questionnaire and the prior knowledge task *via* an online survey tool (unipark), which took about 15 min. Afterwards, in an individual learning session, learners received a pair of headphones and virtual-reality glasses. The participants watched the VR learning unit about seawater desalination, which lasted about 8 min, and was started by the researcher either with the annotations on or off, to prevent technical problems. After finishing the VR learning unit, the participants filled out a post-test online questionnaire including the test of learning outcome and intrinsic motivation while learning, which took around 15 min. At the end of the questionnaire, participants were able to leave feedback about the learning unit and their subjective experience in VR.

## Results

### Descriptive Results

Learners' domain-specific prior knowledge was rather low in both experimental conditions (see Table 1). The extent of intrinsic motivation can be classified as moderate for both groups. The learning outcome was on a medium to a high level, both overall and on the three levels knowledge, comprehension, and application.

**Table 1:** Means and standard deviations of the different experimental conditions.

	**With annotations**	**Without annotations**
	***n* = 28**	***n* = 33**
	***M (SD)***	***M* (*SD*)**
Prior knowledge (%)	23.85 (14.50)	24.45 (14.49)
Intrinsic motivation (*max* = 15)	8.94 (1.60)	8.76 (1.64)
**Learning outcome (%)**		
Overall	64.07 (14.87)	57.20 (23.07)
Knowledge	61.29 (19.00)	53.74 (23.43)
Comprehension	64.20 (24.00)	58.80 (28.60)
Application	70.33 (34.33)	62.67 (31.00)

No differences between the groups in their preconditions could be found (*F* < 1, *p* > 0.203). Multivariate normal distribution was assumed for the relevant variables for each experimental subgroup (*p* > 0.100). Variances were homogenous based on the Bartlett's-test (*p* > 0.060). For calculation, experimental groups were dummy coded and continuous variables were mean centered. Data preparation and analysis were performed using R 3.5.1.

### Effects of Annotations on Learning Outcome

**Figure 2:**
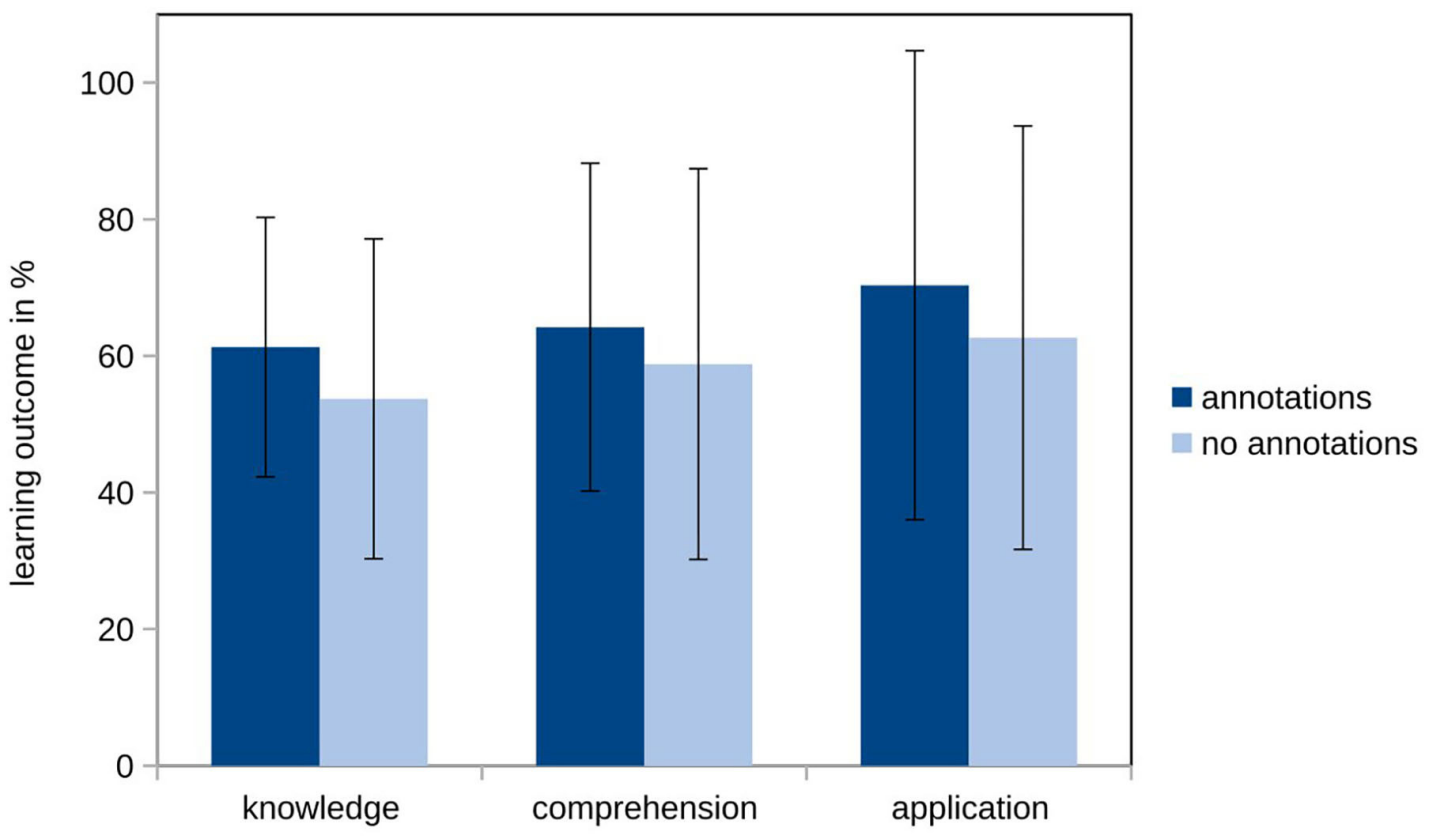
Means and standard deviations of the learning outcome on the three levels knowledge, comprehension and application of the two conditions.

To test our first hypothesis, we analyzed learning outcome based on three of Bloom's (1956) levels. We expected a significant difference between the two experimental groups on all three levels. Analyzing the descriptive pattern of the learning outcome differentiated on the three levels of knowledge, comprehension, and application, we found the expected pattern of our first hypothesis (H1a) on a descriptive level (see Figure 2). The learners who learned with annotations outperformed the learners who learned without annotation.

Despite the descriptive patterns the MANOVA did not reveal the expected significant effect of annotations on learning outcome when subcategorized into knowledge, comprehension, and application (H1a; *F* < 1, *p* = 0.534 $\eta_{partial}^{2}$ = 0.037; see Table 2).

**Table 2:** Results of the ANOVA depending on the level of learning outcome.

	***F*(1, 59)**	***p***	***$\eta_{partial}^{2}$***
Knowledge	1.86	0.177	0.031
Comprehension	0.65	0.425	0.011
Application	0.83	0.366	0.014

Concerning the question of which level of learning processes can be supported best, we compared the effect sizes for the three subcategories, even though none of them were significant (Cohen, 1988). As expected in our hypothesis (H1b), we found the largest effect size for knowledge $\eta_{partial}^{2}$ = 0.031, which can be classified as a small effect (see Table 2). For comprehension and application, we found smaller effect sizes. Even though the descriptive pattern was in line with our expectations, as the experimental groups did not differ significantly in learning outcome (H1a), this hypothesis was not supported by the data.

### Effects of Annotations on Learning Outcome Depending on Learners' Intrinsic Motivation

In our second hypothesis, we expected intrinsic motivation to moderate the relationship between the treatment factor annotations and learning outcome. To test this hypothesis, a hierarchical multiple regression analysis was conducted using the mean centered values. In the first step, we included annotations as the treatment factor in the analysis. Additionally, we included intrinsic motivation as the aptitude factor. As theoretically relevant predictor, we included prior knowledge as a control variable but not as an additional moderator, as this was not the focus of the research question. The first model explained $R_{adj}^{2}$ = 35.89 % of variance in the learning outcome [*F*(3, 57) = 12.20*, p* < 0.001^***^, see Table 3]. In a second step, we added the aptitude-treatment interaction including annotation and intrinsic motivation to the first model. The second model explained $R_{adj}^{2}$ = 41.99% of variance [*F*(4, 56) = 11.86, *f*^2^ = 0.72]. Thus, the interaction accounted for a significant proportion of the variance in the learning outcome (Δ$R_{adj}^{2}$ = 6.10, *p* = 0.011^*^, see Table 3).

**Table 3:** Multiple regression model including prior knowledge, intrinsic motivation, and overall learning outcome.

**Variable**	**Estimate**	**Standard error**	***t*-value**	***p*-value**
**Model 1**				
Intercept	−0.45	0.42	−1.10	0.276
Annotations	1.00	0.62	1.62	0.110
Prior knowledge	0.51	0.11	4.69	<0.001^***^
Intrinsic motivation	0.53	0.20	2.73	0.008
**Model 2**				
Intercept	−0.42	0.39	−1.06	0.295
Annotations	1.01	0.59	1.73	0.090
Prior knowledge	0.47	0.11	4.57	<0.001^***^
Intrinsic motivation	0.97	0.25	3.90	<0.001^***^
Motivation*Annotations	−0.98	0.37	−2.65	0.011^*^

*** *p < 0.001*,

* *p < 0.05. Model 1: ${\text{R}}_{\text{adj}}^{2}$ = 0.36 F_(3, 57)_ = 12.20, p < 0.001; Model 2: ${\text{R}}_{\text{adj}}^{2}$ = 0.42, F_(4, 56)_ = 11.86, p < 0.001*.

**Figure 3:**
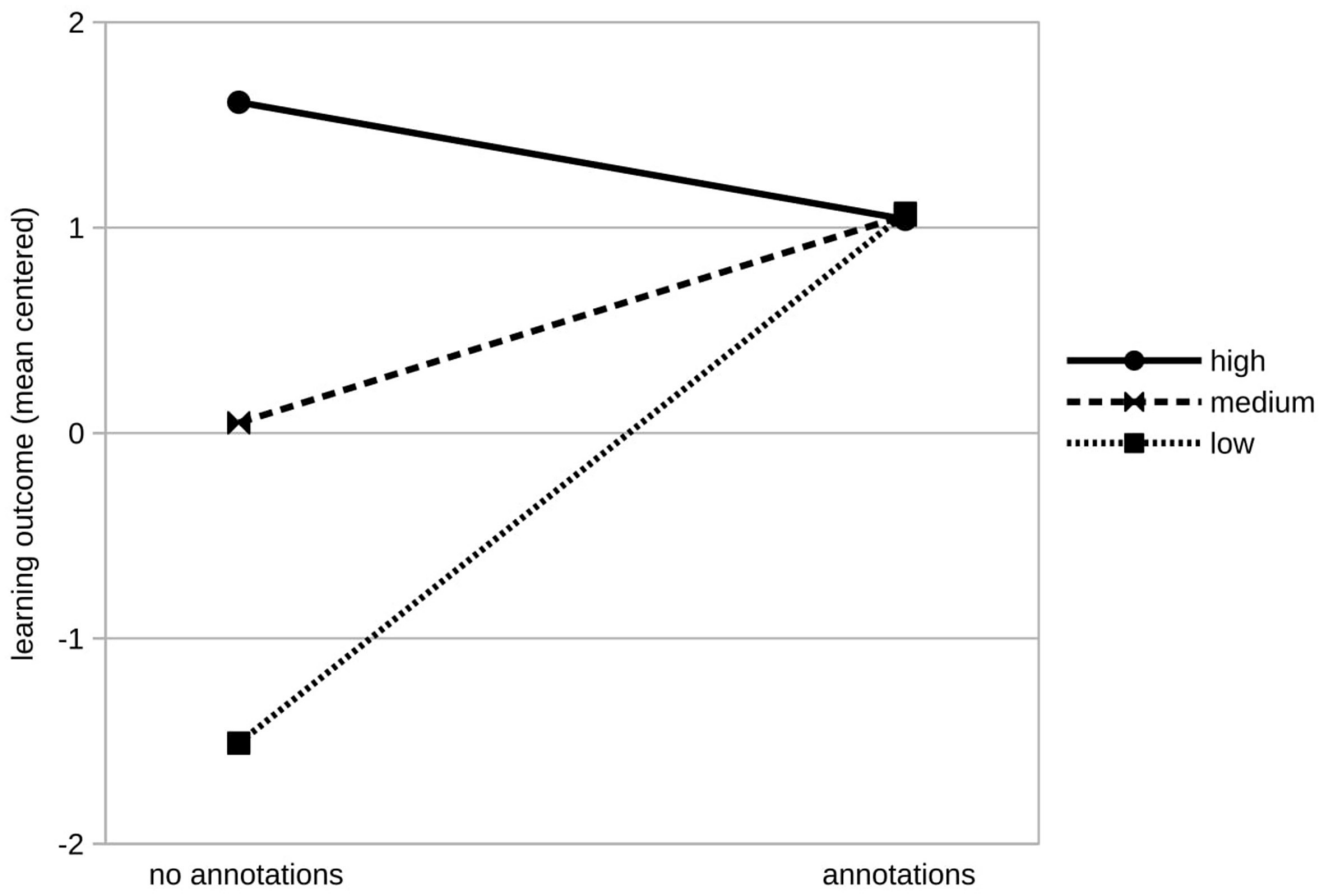
Learning outcome dependent on learners' intrinsic motivation (“low:” one standard deviation below the mean, “medium:” at the mean, and “high” one standard deviation above the mean) and experimental condition.

As expected, prior knowledge had a significant effect on learning outcome (β = 0.47, *SE* = 0.11, *p* < 0.001). The treatment factor had no significant effect on overall learning outcome (β = 1.01, *SE* = 0.59, *p* < 0.090) while the aptitude factor intrinsic motivation had a significant effect (β = 0.97, *SE* = 0.25, *p* < 0.001^***^). We found a significant interaction of annotations and the learners' intrinsic motivation (β = −0.98, *SE* = 0.37, *p* = 0.011^*^). We displayed the moderating effect of intrinsic motivation on learning outcome in Figure 3. To make our result more accessible, we displayed hypothetical groups of learners depending on their intrinsic motivation and experimental group. As recommended by Cohen et al. (2013), we divided learners into three hypothetical groups based on their intrinsic motivation: low (*M*−1 *SD* = −1.61), at the mean (*M* = 0), and high (*M* +1 *SD* = 1.61; see Figure 3). In the control group in which learners had no support by annotations, learners with high intrinsic motivation outperformed those with lower intrinsic motivation. For learners in the conditions with annotations, no substantial effect of intrinsic motivation on the learning outcome was found.

**Figure 4:**
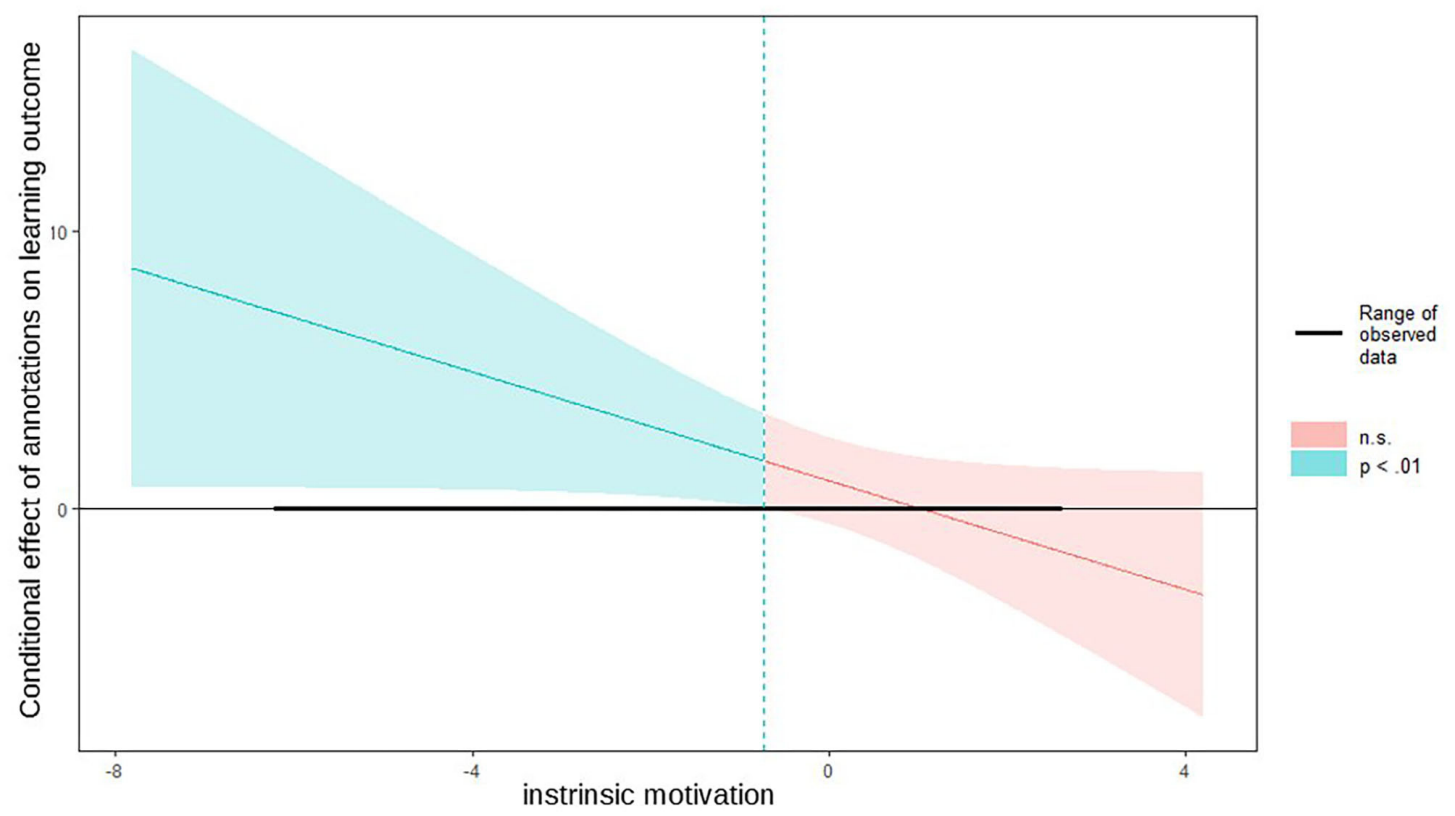
Johnson-Neyman interval for intrinsic motivation moderating the relationship between the treatment factor annotations and learning outcome.

Furthermore, we used the Johnson-Neyman technique (Johnson and Fay, 1950) to determine the exact values of learners' intrinsic motivation, for which the conclusion of significant difference between the experimental groups on the learning outcome can be assumed (see Figure 4). For learners with −0.73 or fewer points on the intrinsic motivation score below the mean, the treatment factors annotations had a significant effect on learning success (*p* < 0.01). Hence, learners need either be intrinsically motivated or need annotations to learn successfully.

### Virtual Reality Experience While Learning

The participants were asked to comment on the experiment and their subjective impressions in the VRLE. Overall, the participants perceived the VRLE as helpful. The design and explanation were explicitly commended as appealing by 23% of the participants. Additionally, 13% of the participants highlighted that their comprehension of the learning content was fostered by the annotations. Around 30% raised concerns about VR and technical issues because the appropriate equipment is needed to gain high-quality output. Furthermore, some participants (15%) mentioned visual strain while being in VR.

## Discussion

While learning complex scientific concepts, learners are usually challenged by integrating multiple representations. This is also the case in a VRLE, where different representations of the learning content, such as pictorial or textual representations, can be displayed. In the present study, we analyzed whether annotations could support these processes and whether these effects depend on learners' motivation.

### Using Annotations to Foster Learning Processes in Virtual Reality

The first hypothesis addressed the effect of annotations on the learning outcome. To gain further insight regarding which cognitive processes are supported best by annotations, we analyzed the effects differentially for the three levels of Bloom (1956): knowledge, comprehension, and application. First, we aimed to answer the question of whether the presence of annotations had a positive effect on the learning outcome on all three levels (H1a). This expectation was not supported by the data. As we predicted in the second part of the first hypothesis, the largest descriptive beneficial effect of annotations was on the knowledge level.

With the merely descriptive, but non-significant, effects, we are in line with previous studies (e.g., Boers et al., 2017). Some previous findings nevertheless described a significant positive effect of annotations on knowledge or recall, but found no effects on higher processing levels, or did not consider them (Mayer and Gallini, 1990; Boers et al., 2017). Other previous findings outline that annotations mainly support deeper cognitive learning processes and learning outcome on the higher levels of comprehension or application (McTigue, 2009; Mason et al., 2013). Taking a closer look at these different results, the question of whether annotations are helpful, and on which level of learning outcome does this beneficial effect occur, depends on the information displayed by the annotation and on the composition of the different representations in the learning material. In the present study, the added annotations were rather simple labels, including one or two words naming relevant components of the learning context. As the labels were placed nearby the corresponding visual information, it also indicated that the corresponding elements could be mapped. However, with this focus on elements, it is plausible that the largest effects have been observed on the knowledge level.

Despite the fact that adding these types of annotations to the VRLE in our present study resulted in a rather small, and not significant, beneficial effect on learning outcome, we carefully outline their impact on the underlying cognitive processes. The positive effects of annotations are assumed to be due to their two functions: signaling and help for element mapping (Gentner et al., 1993; Mayer et al., 1995). The degree to which signaling has a beneficial effect is highly dependent on the visual requirements of the learning material. To uncover a substantial and beneficial effect of annotations, the learning material or environment needs to be sufficiently visually complex (Richter et al., 2016). In the present study, the VRLE consisted of many simplified depictions of the technical or chemical processes. Therefore, the visual display of the learning content was rather a schematic or abstract representation of the learning content. Using abstract representation reduces the unnecessary load while learning, and is hence beneficial (Butcher, 2006). However, this simplification lowers visual requirements, and this might results in lacking the beneficial effects of signaling because they are no longer needed (Richter et al., 2016). As we did not vary the degree of abstraction in our present study, no further assumptions about its influence can be made. Our power analysis was based on a study which also used signaling in a system paced dynamic environment. However, in this study the effect of signaling might be increased as luminance differences were included and by this the effect size of signaling might be enlarged (De Koning et al., 2010).

As outlined earlier, annotations are not *per se* limited to the element layer, but might be extended to foster the relation-to-relation mapping by displaying further information, such as information about related or similar processes of the learning content. Using annotations with relational information might enlarge the positive effect of annotations on the semantic processing and the integration of information into a coherent mental model, and might enlarge the effect on comprehension and application in the learning outcome (Schnotz and Bannert, 2003).

Additionally, in our first hypothesis, we did not consider the learners' aptitudes and states while learning. As learning is an active and resource-consuming process, learners' aptitudes, such as prior knowledge, and the learner's motivational state are crucial for learning success.

### Impact of Annotations and Motivation on Learning Outcome

In our second hypothesis, we analyzed the complex interplay of annotations and intrinsic motivation when controlling for prior knowledge. Learners' prior knowledge as a control variable had the expected large impact, and thus our study is in line with many other studies on expertise-related design effects (Seufert, 2003; Kalyuga, 2009). However, one of the most interesting results of this study is that we found a significant interaction between learners' intrinsic motivation and the presence of annotations. Whereas, learners in the group without annotations differed in their learning outcome depending on their intrinsic motivation, learners in the experimental group with annotations did not show significant differences in their learning outcome depending on their intrinsic motivation. As both factors (intrinsic motivation and signaling through annotations) are described in the literature to have an activating character and a beneficial effect on learning outcomes, our results are in line with these previous findings (Lepper et al., 2005; Richter et al., 2016).

In conclusion, learners are either intrinsically motivated, which results in a higher learning outcome compared to less motivated learners, or they are supported by guiding elements, such as annotations, which reduce visual search while learning. As reflected by the CTML and the CATLM, the learner has an active role while learning (Mayer, 2005; Moreno and Mayer, 2007). Based on the literature, one can assume that when the learner is intrinsically motivated, this goes along with a higher investment of cognitive resources, and this might lead to a higher learning outcome (e.g., Song et al., 2016). As outlined earlier, high intrinsic motivation goes along with more efficient search patterns (e.g., Baranes et al., 2014), and hence learners can compensate for the lack of annotations. Said another way, when less motivated learners received annotations, this also had a beneficial effect on learning, presumably by guiding attention and reducing visual search.

The findings of the present study imply that using annotations in a VRLE has a beneficial effect on the learning outcome, and might bridge the motivation gap by fostering learners who are not intrinsically motivated. But, of course, one could ask why the annotations did not work as an enhancer for motivated students, which would have resulted in even higher scores (i.e., a synergetic effect). In our study, the visual requirements of the VRLE were assumed to be rather low. A twofold activation, leading to “double” investment, seems to have not been necessary. Therefore, a synergetic effect of intrinsic motivation and annotations was neither expected nor found. However, this might be questionable because learners did not reach the maximum of learning outcome in neither of the two activating conditions. Like an illusion of knowing (Avhustiuk et al., 2018), learners might have had the (false) impression that they reached their maximum, and invested sufficient resources. So, one might ask what would have been necessary to activate learners incrementally. Nevertheless, the importance of considering the learners' aptitudes and their current state while learning when analyzing the effects of VRLE was underlined by the present findings.

### Strength and Weaknesses and Recommendations for Future Studies

As the results of the present study indicate, an activating effect of annotations or increased intrinsic motivation on the learner, the underlying mechanisms and cognitive processes need to be further explored. In our present study, the main focus was to uncover the effect of adding annotation and interaction with intrinsic motivation while learning. Moreover, further cognitive mechanisms might be crucial and need to be taken into account. For instance, to uncover the perceived cognitive demands, which are posed by the VRLE, future studies should include a differentiated measurement of cognitive load. Such a measure could help to gain further insight into the distribution of the learners' cognitive resources with regard to intrinsic, extraneous, and germane loading aspects (Klepsch et al., 2017). On the one hand, this would lead to further insights into whether or not additional annotations increase the cognitive demands, particularly the extraneous cognitive load. On the other hand, annotations or motivation might be activating and increase the invested resources of the learner while learning, which would be reflected by germane cognitive load. Additionally, collecting process data *via* eye-tracking or pupillometry might enable deeper insight into the attention guiding effects of annotations and their effects on visual search, as well as the visual requirements of the learning material. Furthermore, the visual search pattern of the integration of information of multiple representations while learning might be investigated by this additional data.

In the present study, we focused on measuring intrinsic motivation. Further aspects of motivation might also be explored in future studies. In our study, motivation has only been treated as a state variable. Motivational traits, for example, goal orientation, might also have a substantial impact on the learning outcome and need to be researched in the field of VRLE (Wolters et al., 1996; Eccles and Wigfield, 2002).

Despite the fact that we found a positive and compensating effect of annotations in VRLEs, the effect might be highly dependent on the concrete design of the learning material, learning content as well as the chosen VRLE. VRLEs range from non-immersive 3D computer simulations to immersive VR with 6 degrees of freedom. Especially in an applied setting such as university courses, the use of simple VR headset like we used in our study would be easier to implement compared to high fidelity VR headsets. However, future studies might investigate the beneficial effects of annotations with other VRLEs. One limitation of this study is that we used a VRLE that neither allows the learners to choose their own perspective nor allows them to interact with the virtual environment. Hence, the field of view was determined. This goes along with the beneficial effect that the challenge of guiding attention in the VRLE does not have to be faced. Therefore, only attentional processes in the field of view need to be considered. When learners are able to choose their own perspective in VR, this leads to additional challenges for the design of VRLEs, and makes it necessary to monitor the learners by using process data during their VR learning session.

The positive effect of annotations for learners with low motivation can therefore not be generalized for all kinds' of VRLEs, as the interactivity in the VR might have an impact on learning outcome. Interaction in VRLEs might also have a beneficial effect on motivational processes, but might cause additional task load or cognitive load, and therefore might be distracting. Hence, further studies are required to gain insights into the potential of VRLEs under consideration of cognitive processes and motivational aspects. Additionally, the setting of the VRLE needs to be considered. In our study, learners used the VRLE in a controlled setting. As uncovered in the pilot study, learners are rather challenged by the handling of the unfamiliar VR-application, and therefore the researcher had to assist with the technical setup. In line with this idea, participants raised many concerns about technical problems in their subjective feedback. Therefore, the question can be raised whether using this kind of learning environment is possible in real-life settings, such as university courses or classroom where the implementation of individual support would be challenging (Checa and Bustillo, 2020). One approach to this challenge would be to train the learners to interact successfully with the technical equipment, and to foster their handling skills in general. This would enhance their ease of use and would foster their technical skills, which could be necessary for future work settings.

## Conclusion

New possibilities to teach scientific concepts in VRLEs come along with the challenge to choose an appropriate design. An adequately designed VRLE supports learners in integrating the learning content into a coherent mental model. For concrete visualization and implementation, learners' aptitudes, for instance, prior knowledge and intrinsic motivation while learning, need to be considered. A lack of intrinsic motivation may be bridged by adding supportive elements, such as annotations. Whereas, highly intrinsically motivated learners seem to successfully face the challenge of integrating information in the mental model, learners who lack intrinsic motivation need to be supported. Future research should focus on uncovering further details about motivational and cognitive processes while learning in VRLEs. Particularly, motivational and cognitive processes, in combination with elements guiding attention and supporting coherence formation, may be essential. One future challenge is to uncover the possibilities of VRLEs and to deduce certain design recommendations and principles for VRLEs.

## Data Availability Statement

The raw data supporting the conclusions of this article will be made available by the authors, without undue reservation.

## Ethics Statement

Ethical review and approval was not required for the study on human participants in accordance with the local legislation and institutional requirements. The patients/participants provided their written informed consent to participate in this study.

## Author Contributions

AV and PA contributed to the conception, design of the studies, developed the used questionnaires, and led data collection for all studies. TS revised the questionnaires. AV analyzed and interpreted the data and drafted the work, which was revised critically by PA and TS. All authors provided approval of the final submitted version of the manuscript, agree to be accountable for all aspects of the work in ensuring that questions related to the accuracy, or integrity of any part of the work are appropriately investigated and resolved.

## Conflict of Interest

The authors declare that the research was conducted in the absence of any commercial or financial relationships that could be construed as a potential conflict of interest.
